# Optimal trade-in strategy for advance selling with strategic consumers proportion

**DOI:** 10.1371/journal.pone.0273124

**Published:** 2023-01-20

**Authors:** Yefeng Wang, Li Zhou, Chuanliang Wu

**Affiliations:** 1 College of International Economics & Trade, Ningbo University of Finance & Economics, Ningbo, Zhejiang, China; 2 University of Greenwich, London, United Kingdom; 3 College of Business, Shanghai University of Finance and Economics, Shanghai, China; Szechenyi Istvan University: Szechenyi Istvan Egyetem, HUNGARY

## Abstract

**Purpose:**

This study aimed to optimize the trade-in pricing strategy. To leverage market share, many sellers adopt trade-in strategy for advance selling, Customers can return their old products at a discount price when they buy new products. This can help increase the market share and decrease natural resource consumption.

**Design/Methodology/Approach:**

We consider a seller who sells new-generation products over two periods: advance selling and regular selling. Based on the rational expectation equilibrium, we adopt dynamic programming to construct a two-period pricing model with three different trade-in strategies–only in period 2, in both periods, and not at all–explaining the trade-in strategy as a promotion tool used by a monopolist to discriminate for advance selling between new and old customers.

**Findings:**

The results suggest that the optimal price is determined by the proportion of old customers, discount factor and product innovation level. Whether and when to give a trade-in rebate to old customers depends on these parameters. The seller’s choice of optimal trade-in strategy depends on the threshold value of the new customer demand and trade-in demand.

**Originality/Value:**

Most existing literature focuses on advance selling strategies and trade-in strategies. To the best of our knowledge, this is a pioneering study that adopts trade-in as part of the advance selling strategy.

## 1. Introduction

Advance selling is a retail practice that a seller accepts customer orders before new products are launched. It has been widely adopted in various industries, such as electronics, fashion, travel and ticketing. For example, customers were encouraged to pre-order Apple smartphone XR before it was released, The advance selling period is also called the first period in this paper; after that, sales became a regular selling period where consumers could purchase in a usual manner, that is, the second period. The benefits of advance selling are threefold: period extension of product sales, more accurate demand forecasting, and improvement in cash flow because customers pay in advance [1].

With the accelerating speed of product updates, an increasing number of customers would replace those “almost new” products with the latest generation products. According to a survey in China, it takes approximately 18 months for customers to switch to a new mobile phone. More than 80 million mobile phones are scrapped every year. Each mobile phone contains over 20 hazardous materials that threaten the environment [2]. The waste of raw materials and pollution from hazardous materials on non-recycled mobile phones is significant. Hence, to achieve the dual goal of sustainability and profitability, some sellers combined the trade-in programs with an advance selling strategy. For instance, Huawei offers its customers a relatively high rebate if they trade-in the last generation of products to buy a newer-generation smartphone.

Most research on advance selling strategies has focused on pricing in the presence of strategic customers without considering trade-in rebates. Other research on trade-in rebates does not consider how the operations, such as refurbishing used product, subside policy, and remanufacturing, could fit into the advance selling strategy. Hence, this study aimed to fill this gap.

To gain a better understanding of (1) whether a seller should implement a trade-in strategy for advance selling; (2) if so, when to adopt trade-in strategy; and (3) how the factors such as strategic consumer behaviour, innovation level, and capacity constraint affect the seller’s optimal trade-in and pricing strategy, we studied three trade-in strategies for advance selling. We developed a conceptual model to reflect customers’ purchasing behaviour and obtain the seller’s decision on the optimal trade-in strategy.

From the seller’s perspective, it is crucial to choose an optimal trade-in strategy that concerns its competitive advantage in the market, that is, no trade-in strategy (strategy 1), providing trade-in strategy only in the second period (strategy 2) and a providing trade-in strategy in both periods (strategy 3)–the advance selling period and the second period. From the customer’s perspective, it is also difficult to decide whether to trade in their old products in the first or second period.

The rest of the paper is organized as follows. Section 2 reviews the relevant literature to position our research and its contributions. The mathematical model is described in Section 3. Section 4 analyzes three trade-in strategies for advance selling and management implications. Section 5 concludes the paper, highlights the contributions and provides direction for future research.

## 2. Relevant literature

There are two main streams of research related to our work: (1) advance selling with strategic consumer behaviour; and (2) trade-in strategy from operations management. Our model bridges the gap between these two distinct streams.

### 2.1 Advance selling strategy with strategic consumers

Existing studies on advance selling have explored various pricing strategies under monopoly [3, 4] or duopoly setting, including manufacturer and retailer [5, 6] or two competitive retailers [1, 7]. Different pricing strategies for advance selling include discount on the pre-order price when customers’ valuation or demands are uncertain [8, 9], the premium pre-order price for a perishable product [10, 11], or the price when the capacity is relatively small [12, 13] and same pre-order price [14]. In addition, some extant literature took advance selling strategy as a price discrimination tool [15] or a competitive marketing instrument [16] to study. Some scholars combined advance selling strategy and financing [17] or contract choice [18] between a supplier and a retailer. Wu et al. [19] combined advance selling and advertising while we combined advance selling and trade-in strategies while between a retailer and consumers. For a detailed review on advance selling strategy literature, we refer the readers to Ma et al. [20] and Wei and Zhang [21].

Many related studies explicitly consider strategic consumer behaviour; customers optimally select their purchasing time to maximize the consuming surplus. Strategic consumers choose to purchase at a lower price in the second period. Strategic consumers negatively influence a seller’s profitability [22, 23]. Therefore, different pricing mechanisms have been proposed to mitigate such negative effect, such as posterior price matching [24, 25], pricing commitment mechanism [26, 27], pricing guarantee mechanism [28, 29] and return guarantee mechanism [13, 30]. However, very few studies have considered trade-in strategy for advance selling that have considered strategic consumers [31]. Table 1 summarises relevant research on strategic consumers.

**Table 1:** Summary of research on pricing mechanisms with strategic customers.

Reference	Capacity	Marketing Size	Customer Type	Customers Valuation	Pricing Mechanism
Lai et al. 2010	Infinite	Infinite	High- and low-end customers	Depreciated	Pricing match mechanism
Zeng 2013	Finite	Infinite	Experienced and inexperienced customers	Constant	Pricing commitment mechanism
Peng et al.2020	Finite	Finite	High-type and low-type customers	Variable with demand	Pricing guarantee mechanism
Nasiry and Popescu 2013	Limited constraint	Infinite	Action regret	Uncertain	Return guarantee mechanism
Liu et al. 2019	Infinite	1	Strategic and myopic customers	Heterogeneous	Trade-in mechanism
This paper	Limited constraint	1	High-and low-end customers	Homogeneous	Trade-in mechanism

In this paper, similar to Liu et al. [31], we consider the existence of rational expectations of strategic consumers. Also, as in Xie and Shugan [12] and Wei and Zhang [21] we consider finite capacity levels, innovation levels, and high and low-value customers’ demand, whilst considering the proportion of trade-in customers.

### 2.2 Trade-in programs

Most trade-in literature focuses on remanufacturing/refurbishing used products [32–34] and subsidy policy [35, 36] for the trade-in strategy in a closed-loop supply chain. Ray et al. [37]examined the value of price discrimination for new and repeat customers of different ages (and product qualities) of the products returned through trade-ins for remanufacturing. Ma et al. [38] studied the impact of a government consumption-subsidy program on a dual-channel closed-loop supply chain. Cohen et al. [39] characterised the impact of demand uncertainty on government subsidies for green technology adoption.

Existing literature on the trade-in rebate of the two periods focused on the regular selling period and clearance selling period of old-and new-generation products, but the effect of trade-in rebate in the advance selling period remains scant. For example, Fudenberg and Tirole [40] studied the monopoly pricing of successive generations of durable products such as computers or an automobiles. The monopolist continues to sell an "older" version of goods and new and higher-quality generations in the second selling period. Zhu et al. [41] analyzed how to identify thresholds that determine trade-in operations in a competitive environment in a two-period planning horizon. Neither study considers strategic consumer behaviour. Van Ackere and Reyniers [42, 43] (1993,1995) analyzed a model of forward-looking customers, and Van Ackere and Reyniers [42] constructed the model that applies to products of any degree of durability, from nondurables to durables. Zhang and Zhang [35] studied how customer purchasing behaviour and remanufacturing efficiency affect economic and environmental values, considering a government subsidy policy in a two-period horizon. The trade-in rebate strategy is adopted just in the second selling period, and there are repeat buyers in period 2.

In contrast to these studies, this study focuses on several trade-in strategies for the advance selling of a new generation product, taking the innovation level, capacity level, trade-in demand into account over two selling periods. There are three different trade-in strategies for the seller to decide. Table 2 summarises the relevant research on the trade-in strategy in the two periods.

**Table 2:** Summary of research on trade-in strategy in two periods.

Reference	Product Type & Second Market	Product Strategy	Customer Behaviour	Pricing Strategy
Fudenberg and Tirole (1998)	Successive generations of a durable Product	Upgrades	No	Intertemporal and static price discrimination
Zhu et al. (2016)	The same generation product	Remanufacturing and government subsidy	No	Duopoly competitive pricing
Van Ackere and Reyniers (1993)	Quasi-durable product	Just Trade-in	Forward-looking	Second-period price discrimination
Van Ackere and Reyniers (1995)	Quasi-durable product	Just Trade-in	Myopic and forward-looking customer	Third-degree price discrimination
Zhang and Zhang. (2018)	First-generation product in period 1, the first and second generation in period 2	Remanufacturing and government subsidy	Strategic	Second-period price discrimination
This paper	Same generation product	Pre-order and trade-in	Strategic and myopic	Second-period price discrimination

The primary goal of this study is to deepen our understanding of trade-in strategies for advance selling. Specially, we analyze the impact of strategic consumer demand on optimal pricing and trade-in strategies. For this purpose, we develop a two-period model in which a profit-maximizing monopoly seller sells new generation products in both periods but may adopt different trade-in strategies. The focus of this study is to integrate the trade-in strategy with advance selling and proposes the optimal trade-in strategy under different conditions. Moreover, while other studies focus on durable products or use the durable factor to measure the durable quality (Zhang and Zhang 2018), we mainly focus on innovative products with short shelf lives and quick updates.

## 3. Model and analysis

### 3.1 Model setup

We consider a monopolistic seller who sells new-generation products to customers over two periods. The first period is the advance selling or pre-order period, and the second period is the regular selling or releasing period. The seller allows all customers to purchase a new-generation product for one unit in any period.

There are two customer segments in the market. Here, we define the “new customers” as those who are first-time buyers and those who hold old generation product but do not trade-in to buy the new-generation product, while “old customers” are those who hold the old product and will trade in to buy the new one [37, 44]. All customers have their valuation for the new-generation product, defined as high *v*_*H*_ and low *v*_*L*_ types of customers, respectively, *v*_*H*_>*v*_*L*_. For new customers, we categorize high-type *v*_*H*_ customers as those who have high value for the new product and arrive in period 1, and low-type *v*_*L*_ customers as those who arrive in period 2 (Li and Zhang 2013). Note, ‘arrive’ doesn’t necessarily mean ‘purchase’. For old customers, we group all of them as high-type customers on the assumption that they are familiar with the brand and have sufficient information to evaluate the product before it is released. This assumption is similar to that of Peng et al. [11] and Li and Xu [44].

For the sake of simplicity, we consider the following scenario and make some necessary assumptions: (a) the market size is 1; (b) no second-hand market exists, no price competition exists between the rebates and other offers; (c) the proportion of new customers is *λ*(0≤*λ*≤1) including *θλ* high-type customers and (1−*θ*)*λ* low-type customers, where *θ*∈[0,1] refers to the proportion of new high-type customers over all new customers; (d) the number of old customers is 1−*λ*. As explained earlier, all old customers are high-type customers; therefore, the total number of high-type customers is *θλ*+(1−*λ*). We assume *λ* follows the generation distribution *F*(⋅) and density *f*(⋅). The resulting distributions of the consumer segments are summarised in Table 3.

**Table 3:** A summary of the distribution of consumer types among the population.

Segment	High type	Low type
New customers, (λ)	*θλ*	(1−*θ*)*λ*
Old trade-in customers, (1−*λ*)	1−*λ*	0

**Fig 1:**
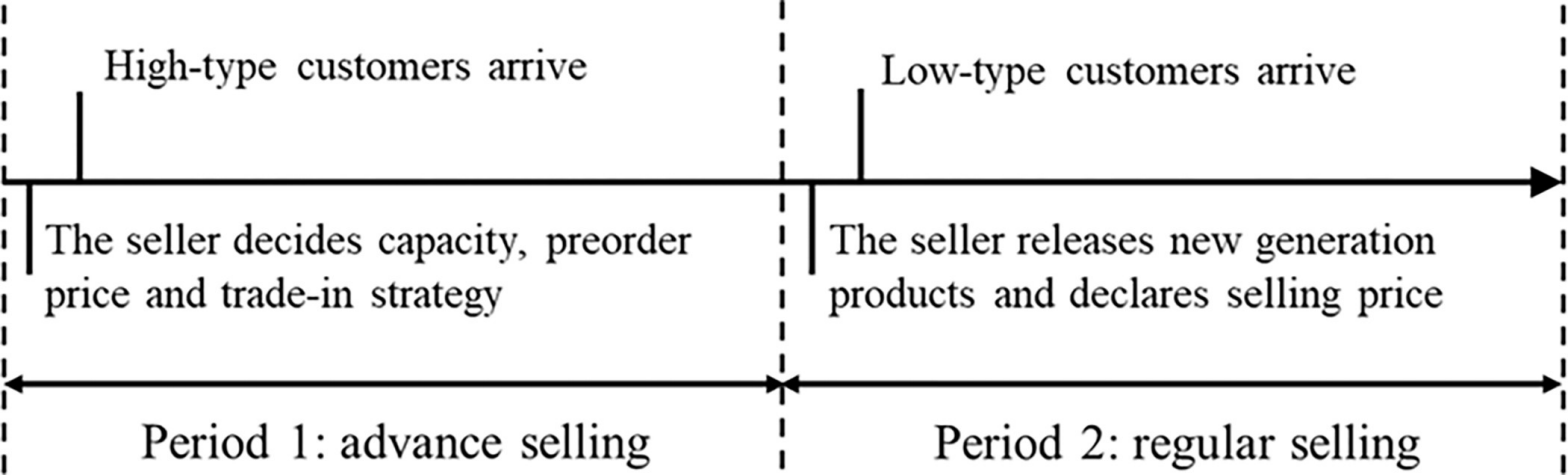
The sequence of events.

We assume that all high-type customers are strategic. There is a risk that this product will be out of stock if strategic consumers wait to buy in the second period. Let *a*(0<*a*≤1) be an exogenously given constant that captures the innovation level, so old customers’ original valuation for the last generation product is $\frac{v_{H}}{1+a}$, let $\beta =\frac{1}{1+a}$, and *βv*_*H*_≤*v*_*L*_ i.e. $0<\beta \leq \frac{v_{L}}{v_{H}}$. This is based on the common sense of both the seller and the customers (see Bala and Carr [45]). Customers are homogeneous ex-ante (i.e. before consuming the product). This is a common setting in models concerning customer purchasing behaviour (see Li and Zhang [4]). The sequence of the events is shown in Fig 1. In the first period, the seller sets the advance selling price *p*_1_ and decides whether to introduce the trade-in strategy. Strategic high-type customers who observe *p*_1_ simultaneously decide to pre-order. In the second period, the seller sets the regular selling price *p*_2_, and all low type customers arrive to buy. We assume that the willingness-to-pay of all high-type customers remains constant in the second period. The seller produces a new generation product at a marginal cost *c*. The seller and customers are risk-neutral and forward-looking. The seller aims to optimize their total expected profit, and the customers maximize their expected net utility. It should be noted that capacity *k*(0<*k*≤1) is a limited constraint.

Following this sequence as shown in Fig 1, we formulate the game in two periods.

To examine the benefit of the trade-in strategy, we consider a benchmark strategy in this study. First, we consider the “base case” in which the seller offers advance selling without a trade-in, i.e. strategy “n”. Second, in addition to the advance selling strategy, we examine the case when the seller offers a trade-in strategy in the second period, i.e. strategy “s”. Finally, the seller offers a trade-in strategy in both periods, i.e. strategy “b”. Throughout this paper, we reserve superscript *i* to denote strategy “*i*”, where $i=n,s,b.\;p_{1}^{i}$ is the pre-order price in the first period and $p_{2}^{i}$ is the regular price in the second period. We assume a discount factor *α* for future periods in different trade-in strategies and assume this to be common to both price and product valuation. Thus, the trade-in price for old customers is $p_{1}^{ri}$ and $p_{2}^{ri}$ in the first and second periods, respectively; $p_{1}^{ri}=\alpha P_{1}^{i},\;p_{2}^{ri}=\alpha P_{2}^{i}$, and *α* is the durable parameter or discount factor for customers’ willingness to pay for old products. Then 1−*α* is a depreciation parameter. The old customers’ valuation for the last-generation product is *αβv*_*H*_ in two selling periods for the new generation product. Then, the old customers’ willingness-to-pay for the new-generation product is *v*_*H*_−*αβv*_*H*_ as adopting a trade-in strategy. Let the trade-in rebate be *r*_*j*_ (*r*_*j*_>*c*) in both periods, *j* = 1,2 denotes the selling period, that is, *r*_1_ = (1−*α*)*P*_1_, *r*_2_ = (1−*α*)*P*_2_. The old (also high-type) customers maintain the original valuation for the new-generation product and the same level of willingness-to-pay as for the last-generation one in both periods. Therefore, whenever the old customers choose a trade-in for the new-generation product, the seller optimally maintains the same trade-in price that is no more than the old high-type customers’ willingness to pay for the last-generation product (see Yin and Li [46]). However, the trade-in rebate will be less in the second period than in the first. $\xi_{1}^{i}$ and $\xi_{2}^{i}$ are the product availability probabilities in the first and second period, respectively. $R_{2}^{i}$ is the seller’s revenue during the second period. *π*^*i*^ is the seller’s total profit in both periods. The unit salvage obtained by subtracting the trade-in cost and dealing with an old last-generation product is *s*_*j*_ (*j* = 1,2) and *s*_1_>*s*_2_≥*c*. The unit salvage must be greater than the trade-in rebate (*s*_*j*_>*r*_*j*_), otherwise, trade-in products have no value to the monopolist.

The decision variable for the seller is pricing strategy. The monopolist sets the pre-order price, regular selling price, and trade-in price.

### 3.2 Benchmark model without trade-in strategy

We adopt the rational expectation (RE) framework to characterize market outcomes. In the RE equilibrium, each player decides based on individual beliefs that are rationally formed and consistent with the actual outcomes. According to the backward induction, we first calculate the optimal regular price and the available probability in the second period when new low-type customers arrive in the second period.

The optimal regular price is $p_{2}^{n*}=v_{L}$. When the seller’s capacity does not exceed the total number of new high-type customers in period 1, i.e. *k*≤*θλ*, the probability of product availability in the second period $\xi_{2}^{n}=0$. When *k*>*θλ* in period 1, the available probability in period 2 is

$$\xi_{2}^{n}=\frac{\mathrm{Emax}[(k-Emin[k,\;\theta \lambda ]),0]}{\lambda -\mathrm{Emin}[k,\theta \lambda ]}=\{\begin{array}{c} F(k)+\int_{k}^{1}\frac{k-\theta \lambda }{(1-\theta )\lambda }d_{F(\lambda )},\;\theta \in [0,k]\\ F(k)+\int_{k}^{\frac{k}{\theta }}\frac{k-\theta \lambda }{(1-\theta )\lambda }d_{F(\lambda )},\theta \in (k,1]) \end{array}$$ (1)

The seller’s expected revenue is $R_{2}^{n}$ in the second period.


$$R_{2}^{n}=p_{2}^{n*}E\min\left[Emax[(k-Emin[k,\theta \lambda ]),0],(\lambda -Emin[k,\theta \lambda ])\right]$$

$$R_{2}^{n}=\{\begin{array}{l} v_{L}\int_{0}^{k}\left(1-\theta \right)\lambda d_{F(\lambda )}+v_{L}\int_{k}^{1}\left(k-\theta \lambda \right)d_{F(\lambda )},\;\theta \in [0,k]\\ v_{L}\int_{0}^{k}\left(1-\theta \right)\lambda d_{F(\lambda )}+v_{L}\int_{k}^{\frac{k}{\theta }}\left(k-\theta \lambda \right)d_{F(\lambda )},\;\theta \in (k,1) \end{array}$$

For new high-type consumers, the utility function is $u_{2}^{n}=(v_{H}-p_{2}^{n*})\xi_{2}^{n}=(v_{H}-v_{L})\xi_{2}^{n}$ in the second period.

All new high-type customers arrive to pre-order the new-generation product in the advance selling period. Because of limited capacity, the product availability probability is $\xi_{1}^{n}$ in the first period.


$$\xi_{1}^{n}=\frac{E\;\min[k,\;\theta \lambda ]}{\theta \lambda }=\{\begin{array}{l} 1\;\theta \in [0,k]\\ F(\frac{k}{\theta })+\int_{\frac{k}{\theta }}^{1}\frac{k}{\theta \lambda }d_{F(\lambda )}\;\theta \in (k,1) \end{array}$$ (2)

According to the RE equilibrium, all strategic high-type customers consistently believe in the reality of the available probabilities and prices in both periods. The utility function is $u_{1}^{n}=(v_{H}-p_{1}^{n})\xi_{1}^{n}$ for high-type customers in the first period. If and only if $u_{1}^{n}=u_{2}^{n}$, high-type customers will pre-order, then, the optimal pre-order price is decided by

$$p_{1}^{n*}=v_{H}-(v_{H}-v_{L})\frac{\xi_{2}^{n}}{\xi_{1}^{n}}$$ (3)

**Proposition 1**. Under the no trade-in strategy for advance selling, there is a unique RE equilibrium in which all new high-type customers pre-order in the first period at the optimal price given by Eq (3).

According to Proposition 1, the optimal pre-order price is decided by the availability of the product in both periods, in particular, the proportion of new high-type customers will affect the product availability probability in the first period. The more high-type customers there are, the higher pre-order price the seller can set.

The firm’s expected total profit is

$$\pi^{n}=p_{1}^{n*}E\;\min\left[k,\theta \lambda \right]+R_{2}^{n}-ck$$

$$\pi^{n}=\{\begin{array}{ll} p_{1}^{n*}\int_{0}^{1}\theta \lambda d_{F(\lambda )}+R_{2}^{n}-ck, & \theta \in [0,k]\\ p_{1}^{n*}\int_{0}^{\frac{k}{\theta }}\theta \lambda d_{F(\lambda )}+p_{1}^{n*}\int_{\bar{a}}^{1}kd_{F(\lambda )}+R_{2}^{n}-ck, & \theta \in (k,1) \end{array}$$ (4)

### 3.3 Advance selling and trade-in strategy in second period

This section investigates the trade-in value in the second period. To begin with backward induction, we calculate the available probability when all new low-type and old high-type customers arrive in the second period.

If the optimal release price is $p_{2}^{s*}=v_{L}$, then the trade-in price is $p_{2}^{rs}=\alpha p_{2}^{s*}=\alpha v_{L}$, and the trade-in rebate is $r=P_{2}^{s*}-P_{2}^{rs}=(1-\alpha )v_{L}$.

For old customers holding used last-generation product, only if trade-in price $\alpha P_{2}^{s*}$ is no more than the willingness to pay for the new-generation product, *v*_*H*_−*αβv*_*H*_, will they adopt a trade-in strategy. The utility function is $\left[(v_{H}-\alpha \beta v_{H})-\alpha P_{2}^{s*}\right]$. In other words, the seller should set $\alpha P_{2}^{s*}\leq v_{H}-\alpha \beta v_{H}$, the maximum trade-in discount factor is $\alpha^{s}\leq \frac{v_{H}}{v_{L}+\beta v_{H}}=\frac{v_{H}}{v_{L}+\frac{v_{H}}{1+a}}$.

When *k*≤*θλ* in period 1, i.e. the capacity is less than demand, there will be no new product to sell in period 2. Then the seller does not need to adopt a trade-in strategy. If *θλ*<*k*<*λ* in period 1, the seller will adopt advance selling without a trade-in strategy because he always firstly satisfies all new customers’ demand to enlarge the market share. The seller executes a trade-in strategy in the second period only when *k*>*λ*. Therefore, the product availability probability in the second period is

$$\xi_{2}^{S}=\frac{\mathrm{Emax}[(k-E\min[k,\theta \lambda ]),0]}{1-\mathrm{Emin}[k,\theta \lambda ]}=\{\begin{array}{l} \int_{0}^{1}\frac{k-\theta \lambda }{1-\theta \lambda }d_{F(\lambda )}\;\theta \in [0,k]\\ \int_{0}^{\frac{k}{\theta }}\frac{k-\theta \lambda }{1-\theta \lambda }d_{F(\lambda )}\;\theta \in (k,1) \end{array}$$ (5)

The utility function of high-type customers is $u_{2}^{s}=(v_{H}-p_{2}^{s*})\xi_{2}^{s}=(v_{H}-v_{L})\xi_{2}^{s}$ in the second period. Then the expected revenue is from the old trade-in and new low-type customers in the second period.


$$R_{2}^{s}=v_{L}E\;\min\left[Emax[(k-Emin[k,\theta \lambda ]),0],(1-Emin[k,\theta \lambda ])\right]+(s_{2}-r_{2})Emin[k-\min\left[k,\lambda \right]]$$

$$R_{2}^{S}=\{\begin{array}{ll} v_{L}\int_{0}^{1}(k-\theta \lambda )d_{F(\lambda )}+(s_{2}-r_{2})\int_{k}^{1}(k-\lambda )d_{F(\lambda )} & \theta \in [0,k]\\ v_{L}\int_{0}^{\frac{k}{\theta }}(k-\theta \lambda )d_{F(\lambda )}+(s_{2}-r_{2})\int_{k}^{\frac{k}{\theta }}(k-\lambda )d_{F(\lambda )} & \theta \in (k,1] \end{array}$$

All new and strategic high-type customers arrive and pre-order new-generation products in the first period. The available probability is $\xi_{1}^{s}$ in the first period,

$$\xi_{1}^{s}=\frac{E\min[k,\;\theta \lambda ]}{\theta \lambda }=\{\begin{array}{ll} 1, & \theta \in [0,k]\\ F(\frac{k}{\theta })+\int_{\frac{k}{\theta }}^{1}\frac{k}{\theta \lambda }d_{F(\lambda )}, & \theta \in (k,1] \end{array}$$ (6)

According to the RE equilibrium, for all new high-type customers, the utility function is $u_{1}^{s}$ in the first period, then $u^{s}=(v_{H}-p_{1}^{s})\xi_{1}^{s}$.

If and only if $u_{1}^{s}=u_{2}^{s}$, high-type customers will pre-order, then,

$(v_{H}-p_{1}^{s})\xi_{1}^{s}=(v_{H}-v_{L})\xi_{2}^{s}$, we obtain the optimal pre-order price for the proportion of new high-type customers.


$$p_{1}^{s*}=v_{H}-(v_{H}-v_{L})\frac{\xi_{2}^{s}}{\xi_{1}^{s}}$$ (7)

**Theorem** 1: Under a single trade-in strategy for advance selling, (ⅰ) the optimal pre-order price has nothing to do with the innovation level. (ⅱ) $P_{1}^{s*}$ are strictly increased with *θ*.

**Proposition 2**. The seller can set up the optimal pre-order price and trade-in price is $p_{1}^{s*}$ and *αv*_*L*_ respectively, the optimal trade-in rebate is (1−*α*)*v*_*L*_. The old high-type customers would like to use the trade-in rebate to buy the new-generation one in the second period when the trade-in discount factor satisfies the condition with $0\leq \alpha \leq \frac{v_{H}}{v_{L}+\frac{v_{H}}{1+a}}$, that is, there exists a threshold value $\bar{\alpha }=\frac{v_{H}}{v_{L}+\frac{v_{H}}{\left(1+a\right)}}$. The trade-in price $P_{2}^{rs}$ strictly increases with the innovation level *a*. The trade-in rebate strictly decreases with the innovation level *a*.

In Proposition 2, we find that the ceiling of the trade-in discount factor is determined by the innovation parameter. When the innovation parameter is higher, the old high-type customers would rather buy the new-generation product at a higher trade-in price than hold the old one. Then, the seller can decide a higher trade-in price.

With trade-in strategy just in the second period, the seller’s expected total profit is

$$\pi^{s}=p_{1}^{s*}Emin[k,\theta \lambda ]+R_{2}^{s}-ck$$

$$\pi^{S}=\{\begin{array}{ll} p_{1}^{S*}\int_{0}^{1}\theta \lambda d_{F(\lambda )}+R_{2}^{S}-ck, & \theta \in [0,k]\\ p_{1}^{S*}\int_{0}^{\frac{k}{\theta }}\theta \lambda d_{F(\lambda )}+p_{1}^{S*}\int_{\frac{k}{0}}^{1}kd_{F(\lambda )}+R_{2}^{S}-ck, & \theta \in (k,1) \end{array}$$ (8)

### 3.4 Advance selling and trade-in strategy in both periods

If the optimal release price is still $p_{2}^{b*}=v_{L}$ in the second period, then the trade-in price is $p_{2}^{rb}=\alpha p_{2}^{b*}=\alpha v_{L}$ in period 2, and the trade-in rebate is $r=P_{2}^{b*}-P_{2}^{rb}=(1-\alpha )v_{L}$.

When the seller permits old customers to trade-in in either period, all old high-type customers would certainly choose to use the trade-in rebate to pre-order in the first period because they are strategic. When the seller’s capacity is less than the demand for new and old high-type customers, that is, *k*≤1−*λ*+*θλ*, the product availability probability is $\xi_{2}^{b}=0(0\leq \lambda <\frac{1-k}{1-\theta }$) in the second period. Thus, the revenue is $R_{2}^{b}=0$ in period 2. The available probability is $\xi_{1}^{b}=\frac{k}{1-(1-\theta )\lambda }$ in period 1. The seller does not need to adopt a trade-in strategy in the second period because of no capacity. Therefore, as $0\leq \lambda <\frac{1-k}{1-\theta }$, the seller adopts a pre-order and trade-in strategy in period 1.

When 1−*λ*+*θλ*<*k*<1, that is. $\frac{1-k}{1-\theta }<\lambda \leq 1$ and *θ* satisfies the condition 0≤*θ*<*k*, which means the seller’s capacity must be so large that the trade-in strategy is worth implementing, then the product availability probability in period 2 is

$$\xi_{2}^{b}=\frac{Emax[(k-E\min\left[k,\theta \lambda +(1-\lambda )]),0\right]}{1-Emin[k,\theta \lambda +(1-\lambda )]}=\int_{\frac{1-k}{1-\theta }}^{1}\frac{k-[1-\lambda +\theta \lambda ]}{(1-\theta )\lambda }d_{F(\lambda )},\;\theta \in [0,k]$$ (9)

Then the expected revenue in period 2 is,

$${R_{2}^{bs}=v}_{L}E\;\min\left[Emax[(k-Emin[k,\theta \lambda +(1-\lambda )]),0],(1-Emin[k,\theta \lambda +(1-\lambda )])\right]$$

$$R_{2}^{b}=\{\begin{array}{ll} v_{L}\int_{\frac{1-k}{1-\theta }}^{1}(k-(1-\lambda +\theta \lambda ))d_{F(\lambda )}, & \theta \in [0,k].\\ 0 & \theta \in (k,1) \end{array}$$

For new low-type customers, the utility function in the second period is

$$u_{2}^{b}=(v_{H}-p_{2}^{b*})\xi_{2}^{b}=(v_{H}-v_{L})\xi_{2}^{b}.$$

The availability probability is $\xi_{1}^{b}$ in the first period, then

$$\xi_{1}^{b}=\frac{E\;\min[k,1-\lambda +\theta \lambda ]}{1-\lambda +\theta \lambda }=\{\begin{array}{ll} \int_{0}^{\frac{1-k}{1-\theta }}\frac{k}{1-\lambda +\theta \lambda }d_{F(\lambda )}+\int_{\frac{1-k}{1-\theta }}^{1}1d_{F(\lambda )}, & \theta \in [0,k]\\ \int_{0}^{1}\frac{k}{1-\lambda +\theta \lambda }d_{F(\lambda )}, & \theta \in (k,1) \end{array}$$ (10)

The utility function is $u_{1}^{b}$ for high-type customers in the first period, then $u_{1}^{b}=(v_{H}-p_{1}^{b})\xi_{1}^{b}$. If and only if $u_{1}^{b}=u_{2}^{b}$, all high-type strategic customers will pre-order, then, $(v_{H}-p_{1}^{b})\xi_{1}^{b}=(v_{H}-v_{L})\xi_{2}^{b}$. Thus, the optimal pre-order price is in the first period.


$$p_{1}^{b*}=v_{H}-(v_{H}-v_{L})\frac{\xi_{2}^{b}}{\xi_{1}^{b}}$$ (11)

Then the trade-in price is $\alpha p_{1}^{b*}$, that is, $p_{1}^{rb}=\alpha p_{1}^{b*}$, and the trade-in rebate is $r_{1}=(1-\alpha )p_{1}^{b*}$ in the first period.

The expected revenue is $R_{1}^{b}$ in the first period.


$$R_{1}^{b}=P_{1}^{b*}E\min\left[k,(1-\lambda +\theta \lambda )\right]+(s_{1}-r_{1})Emin[[k-E\min\left[k,\theta \lambda \right]],(1-\lambda )]$$

$$\begin{array}{l} P_{1}^{b*}\int_{0}^{\frac{1-k}{1-\theta }}kd_{F(\lambda )}+P_{1}^{b*}\int_{\frac{1-k}{1-\theta }}^{1}(1-\lambda +\theta \lambda )d_{F(\lambda )}+(s_{1}-r_{1})\int_{0}^{\frac{1-k}{1-\theta }}(k-\theta \lambda )d_{F(\lambda )}+(s_{1}-r_{1})\int_{\frac{1-k}{1-\theta }}^{1}(1-\lambda )d_{F(\lambda )},\;\theta \in [0,k]\\ R_{1}^{b}=P_{1}^{b*}\int_{0}^{1}kd_{F(\lambda )}++(s_{1}-r_{1})\int_{0}^{\frac{k}{\theta }}(k-\theta \lambda )d_{F(\lambda )},\;\theta \in (k,1) \end{array}$$

The seller’s expected total profit is

$$\pi^{b}=R_{1}^{b}+R_{2}^{b}-ck$$ (12)

Only if the trade-in price $\alpha P_{1}^{b*}$ is no more than *v*_*H*_−*α*⋅*βv*_*H*_, the old customer would trade-in. At least the seller should set up $\alpha P_{1}^{b*}\leq v_{H}-\alpha \cdot \beta v_{H}$, i.e. $\alpha \leq \frac{v_{H}}{P_{1}^{b*}+\beta v_{H}}$. The optimal trade-in discount factor, $\alpha^{*}=\frac{1}{1+\beta }$.

When 0≤*θ*<*k*, the ceiling of the trade-in discount factor is less than $\frac{v_{H}}{v_{L}+\beta v_{H}}$ of the trade-in strategy in the second period; therefore, the seller needs to set up a lower trade-in price to induce more old high-type customers to pre-order in the first period when the proportion of new high-type customers is not sufficiently large.

The purchasing utility of old customers is $[(v_{H}-\alpha \cdot \beta v_{H})-\alpha \cdot P_{1}^{b*}]\cdot \xi_{1}^{b}$ and $[(v_{H}-\alpha \cdot \beta v_{H})-\alpha \cdot P_{2}^{b*}]\cdot \xi_{2}^{b}$ in the first and second periods, respectively.

To induce the old customers to trade-in as early as possible rather than wait until the second period, according to $u_{1}^{rb}=u_{2}^{rb}$ for old customers,

$$[(v_{H}-\alpha \cdot \beta v_{H})-\alpha \cdot P_{1}^{b*})\cdot \xi_{1}^{b}=[(v_{H}-\alpha \cdot \beta v_{H})-\alpha \cdot P_{2}^{b*}]\cdot \xi_{2}^{b}$$

Then we can induce the optimal trade-in discount factor to be $\alpha^{*}=\frac{(\xi_{1}^{b}-\xi_{2}^{b})\cdot v_{H}}{(\beta v_{H}+P_{1}^{b*})\cdot \xi_{1}^{b}-(\beta v_{H}+v_{L})\cdot \xi_{2}^{b}}=\frac{1}{1+\beta }$ for 0≤*θ*<*k*

Either 0≤*θ*<*k* or *k*≤*θ*<1, the optimal trade-in discount factor is the same.

**Property 3**. We find (ⅰ) there is only one optimal pre-order price as 0≤*θ*<*k*. And the optimal trade-in discount factor exists $\alpha^{*}=\frac{1}{1+\beta }$, which means the discount factor is only related to the innovation level *a*. Recall $\beta =\frac{1}{1+a}$. (ⅱ) the optimal pre-order price is strictly increasing in *θ* as 0≤*θ*<*k*.

For 0≤*θ*<*k*, when all high-type customers including all old customers using the trade-in rebate pre-order in the first period, and all low-type customers buy in the second period, the seller’s expected total profit is

$$\begin{array}{l} \pi^{b}=p_{1}^{b*}Emin[k,1-\lambda +\theta \lambda ]+R_{2}^{b}-(r_{1}-s_{1})(1-\lambda )-ck\\ \;=P_{1}^{b*}\int_{0}^{\frac{1-k}{1-\theta }}kd_{F(\lambda )}+P_{1}^{b*}\int_{\frac{1-k}{1-\theta }}^{1}{(1-\lambda +\theta \lambda )d}_{F(\lambda )}+(s_{1}-r_{1})\int_{0}^{\frac{1-k}{1-\theta }}(k-{\theta \lambda )d}_{F(\lambda )}+(s_{1}-r_{1})\int_{\frac{1-k}{1-\theta }}^{1}(1-{\lambda )d}_{F(\lambda )}+R_{2}^{b}-ck, \end{array}$$

(0≤*λ*<*k*)

Since the availability probability is zero as *k*≤*θ*≤1, it is beneficial for the seller to implement the trade-in strategy for advance selling just in the condition of 0≤*θ*<*k*, which means the seller should induce more old high-type customers to pre-order in the first period when new high-type customer’ demand is not sufficiently large.

## 4. The performance of different trade-in program for advance selling strategy

### 4.1 the seller’s optima pricing strategy

(i) 0≤*θ*<*k*

It is obviously that $\xi_{1}^{s}=\xi_{1}^{n}=1,\xi_{2}^{s}<\xi_{2}^{n}$, so $p_{1}^{s*}>p_{1}^{n*}$ for 0≤*θ*<*k*.And we assume that the new consumers demand *λ* is uniform distribution in the distance [0, 1]. Then, we can obtain the available probability in different strategies.

Because of $\xi_{1}^{b}<\xi_{1}^{s}=\xi_{1}^{n}=1,\xi_{2}^{b}<\xi_{2}^{s}<\xi_{2}^{n}$, So we compare $p_{1}^{s*}$ with $p_{1}^{b*}$

$$\frac{\xi_{2}^{b}}{\xi_{1}^{b}}-\frac{\xi_{2}^{s}}{\xi_{1}^{s}}=-1-\frac{1-k}{\theta }\ln\left(\frac{1-k}{1-\theta }\right)+\frac{(k-\theta )+(1-k)\ln\left(\frac{1-k}{1-\theta }\right)}{\left(1-k+k\cdot \ln(1-k+\theta \right)}<0$$

$$p_{1}^{b*}-p_{1}^{s*}=-(v_{H}-v_{L})(\frac{\xi_{2}^{b}}{\xi_{1}^{b}}-\frac{\xi_{2}^{s}}{\xi_{1}^{s}})>0$$

According to $p_{1}^{s*}>p_{1}^{n*}$ for 0≤*θ*<*k*, we can get property 4.

**Property 4**. Comparing the Eqs (3) with (7), we know the pre-order price for no trade-in is always less than one for advance selling with just trade-in program in the second period as 0≤*θ*<*k*. This implies that $p_{1}^{s*}>p_{1}^{n*}$. We can obtain the result $p_{1}^{b*}>p_{1}^{s*}>p_{1}^{n*}$ according to the Eqs (7) with (11), and $\frac{\xi_{2}^{b}}{\xi_{1}^{b}}-\frac{\xi_{2}^{s}}{\xi_{1}^{s}}<0$

According to the Property 4, the seller can always set up highest pre-order price with the trade-in program in both periods as 0≤*θ*<*k*. This result is consistent with practice. The seller can use trade-in program in both periods to induce old and new high-type consumers to obtain a higher pre-order price.

(ii) *k*≤*θ*≤1

When the proportion of new high-type customers is no less than the capacity number, *k*≤*θ*≤1, it’s useless for the seller to adopt the trade-in program in both periods for advance selling strategy. Therefore, we analyze only the two strategies ahead, *i* = *n*, *b*. We find that $p_{1}^{n*}>P_{1}^{s*}$ because of $\xi_{2}^{n}>\xi_{2}^{s}$ according to (1) and (5), $\xi_{1}^{n}=\xi_{1}^{s}$. Combining situation 3.3, we find the seller can set up the highest pre-order price $p_{1}^{b*}=v_{H}$ with advance selling of both trade-in programs because $\xi_{2}^{b}=0$ as *k*≤*θ*≤1.

**Theorem 2.** According to Eqs (2) and (6), (3) and (7), the pre-order price increases when the proportion of new consumers is even larger than the numerical value of the capacity. i.e. $v_{H}=p_{1}^{b*}>p_{1}^{s*}>P_{1}^{n*}$ because of $\xi_{2}^{b}=0$ as *k*≤*θ*≤1.

According to Theorem 2, the seller can set up highest pre-order price $p_{1}^{b*}=v_{H}$ with the increase in the proportion of new high-type consumers with the trade-in program in both periods than no trade-in and only in the second period for advance selling, because of the old high-type consumers arriving in the first period.

### 4.2 the seller’s optimal profits

(i) 0≤*θ*<*k*


$$\pi^{s}-\pi^{n}=(P_{1}^{s*}-P_{1}^{n*})\int_{0}^{1}\theta \lambda d_{F(\lambda )}+(s_{2}-r_{2})\int_{k}^{1}\left(k-\lambda \right)d_{F(\lambda )}{-v}_{L}\int_{0}^{k}\left(k-\lambda \right)d_{F(\lambda )}=\frac{(P_{1}^{s*}-P_{1}^{n*})\theta +(1-\alpha ){{\left(1-k\right)}^{2}v}_{L}-s_{2}{\left(1-k\right)}^{2}{-v}_{L}k^{2}}{2}\alpha \leq \frac{\theta (P_{1}^{s*}-P_{1}^{n*})+(v_{L}-s_{2}){\left(1-k\right)}^{2}{-v}_{L}k^{2}}{{{\left(1-k\right)}^{2}v}_{L}}$$

*π*^*s*^*−π*^*n*^≥0

This means that it is always beneficial for the seller only if the durable parameter or discount factor for the customers’ willingness to pay for old products is no more than its threshold.


$$\pi^{b*}-\pi^{n*}=P_{1}^{b*}\int_{0}^{\frac{k-\theta }{1-\theta }}{kd}_{F(\lambda )}+P_{1}^{b*}\int_{\frac{k-\theta }{1-\theta }}^{1}{(1-\lambda +\theta \lambda )d}_{F(\lambda )}-p_{1}^{n^{'}*}\int_{0}^{1}{\theta \lambda d}_{F(\lambda )}+(s_{1}-r_{1})\int_{0}^{\frac{1-k}{1-\theta }}(k-{\theta \lambda )d}_{F(\lambda )}+(s_{1}-r_{1})\int_{\frac{1-k}{1-\theta }}^{1}(1-{\lambda )d}_{F(\lambda )}+v_{L}\int_{\frac{1-k}{1-\theta }}^{1}\left(k-(1-\lambda +\theta \lambda )\right)d_{F(\lambda )}-v_{L}\int_{0}^{k}(1-\theta )\lambda d_{F(\lambda )}-v_{L}\int_{k}^{1}\left(k-\theta \lambda \right)d_{F(\lambda )}$$

The forehead five items delegate the first profit difference, the last three items delegate the second profit difference. The second profit difference is positive.


$$\alpha \geq 1-\frac{s_{1}}{P_{1}^{b*}}-\frac{P_{1}^{b*}\int_{0}^{\frac{k-\theta }{1-\theta }}{kd}_{F(\lambda )}+P_{1}^{b*}\int_{\frac{k-\theta }{1-\theta }}^{1}{(1-\lambda +\theta \lambda )d}_{F(\lambda )}-p_{1}^{n^{'}*}\int_{0}^{1}{\theta \lambda d}_{F(\lambda )}}{P_{1}^{b*}(\int_{0}^{\frac{1-k}{1-\theta }}(k-{\theta \lambda )d}_{F(\lambda )}+\int_{\frac{1-k}{1-\theta }}^{1}(1-{\lambda )d}_{F(\lambda )})}$$

$$-\frac{v_{L}\int_{\frac{1-k}{1-\theta }}^{1}\left(k-(1-\lambda +\theta \lambda )\right)d_{F(\lambda )}-v_{L}\int_{0}^{k}(1-\theta )\lambda d_{F(\lambda )}-v_{L}\int_{k}^{1}\left(k-\theta \lambda \right)d_{F(\lambda )}}{P_{1}^{b*}(\int_{0}^{\frac{1-k}{1-\theta }}(k-{\theta \lambda )d}_{F(\lambda )}+\int_{\frac{1-k}{1-\theta }}^{1}(1-{\lambda )d}_{F(\lambda )})}$$

To guarantee ${\pi^{b*}-\pi }^{n*}\geq 0$ is always true, we can induce $\theta \geq \frac{\lambda P_{1}^{b*}}{(1-\lambda )P_{1}^{n*}}$, so the number of new high-type consumers can not be too small. Otherwise, the trade-in program for advance selling in both periods is not optimal and will hurt the seller’s benefit.

(ii) *k*≤*θ*≤1

We then analyze the different profits in the three different strategies. According to Eqs (4) and (8), we can calculate the profit difference as

$${\pi^{s}-\pi }^{n}={(p}_{1}^{s*}-p_{1}^{n*})(\int_{0}^{\frac{k}{\theta }}\theta \lambda d_{F(\lambda )}+\int_{\frac{k}{\theta }}^{1}kd_{F(\lambda )})+{v_{L}\int_{0}^{k}\left(k-\theta \lambda \right)d_{F(\lambda )}+(s_{2}-r_{2})\int_{k}^{\frac{k}{\theta }}\left(k-\lambda \right)d_{F(\lambda )}-v_{L}\int_{0}^{k}(1-\theta )\lambda d_{F(\lambda )}}_{}^{}$$

*π*^*s*^−*π*^*n*^≥0, as

$$\alpha \geq 1-\frac{s_{2}}{v_{L}}-\frac{(v_{H}-v_{L})\frac{\xi_{2}^{n}-\xi_{2}^{s}}{\xi_{1}^{n}}(\frac{2k\theta -k^{2}}{2\theta })+v_{L}\int_{0}^{k}\left(k-\lambda \right)d_{F(\lambda )}}{v_{L}\int_{k}^{\frac{k}{\theta }}\left(k-\lambda \right)d_{F(\lambda )}}>0$$

$$\underline{\alpha }=1-\frac{s_{2}}{v_{L}}-\frac{(\frac{v_{H}}{v_{L}}-1)\frac{\xi_{2}^{n}-\xi_{2}^{s}}{\xi_{1}^{n}}(2\theta -k)-k\cdot \theta^{2}}{(1-\theta^{2})\cdot k}$$

We find that *α* increases with the new high-type customers’ demands. When new high-type customers’ demands are higher, the seller can control higher trade-in price, lower trade-in rebate.

Because the unit revenue from dealing with the old product is certainly more than that of the trade-in rebate, the profit difference is more than zero. This means that it is optimal for a seller to adopt trade-in program for any *θ*.

**Property 5**. For *k*<*θ*≤1, the seller can use trade-in for advance selling in both periods, but the trade-in price must be larger than $\underline{\alpha }\cdot P_{2}^{s*}$, and the trade-in discount factor must lie in the district $\left[\underline{\alpha },\bar{\alpha }\right]$. Otherwise, the seller can adopt trade-in program in second period for advance selling strategy.

## 5. Comparison of the seller’s optimal strategy–a numerical example

We set *k* = 0.85, *v*_*H*_ = 3.0, *v*_*L*_ = 2.0, *c* = 0.1, *s*_1_ = 0.25, *s*_2_ = 0.15, *β* = 0.667 (as *a* = 0.5), we can calculate α* = 0.5999.

### 5.1 Optimal pre-order price of different trade-in strategies

**Fig 2:**
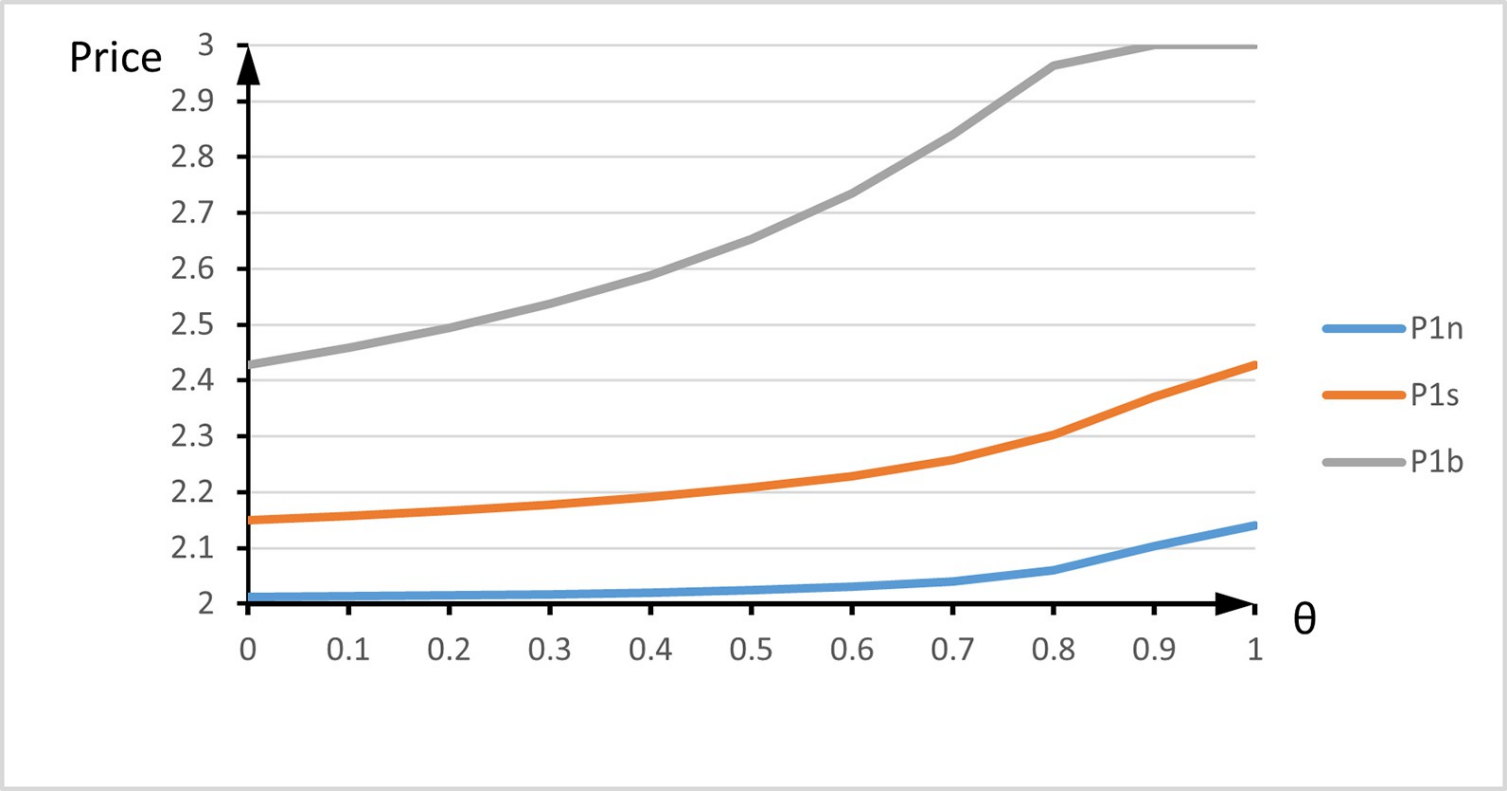
the influence of new high-type customers number on optimal pre-order price for different trade-in strategies.

Fig 2 elaborates on the optimal pricing strategy, where *θ*-axis is the demand of all new high-type customers, and the price-axis is the seller’s optimal prices under different trade-in strategies. Fig 2 suggests that the optimal pre-order price $P_{1}^{n}$ and $P_{1}^{s}$ strictly increase with *θ* under no trade-in strategy and trade-in strategy in period 2. Fig 2 shows that under trade-in strategy in both periods, $P_{1}^{b}$ increases with the quantity of all new high-type customers for 0≤*θ*<*k* and remains constant and the same as the customer’s high value for the new generation product for *k*<*θ*≤1.Fig 2 illustrates that under both trade-in strategies, the optimal pre-order price is the largest, $P_{1}^{b}>P_{1}^{s}>P_{1}^{n}$. Hence, the more new high-type customers, the higher pre-order price. This makes sense from the marketing perspective. A high *θ* indicates the popularity of the new-generation products.

### 5.2 The profit of different trade-in strategies

**Fig 3:**
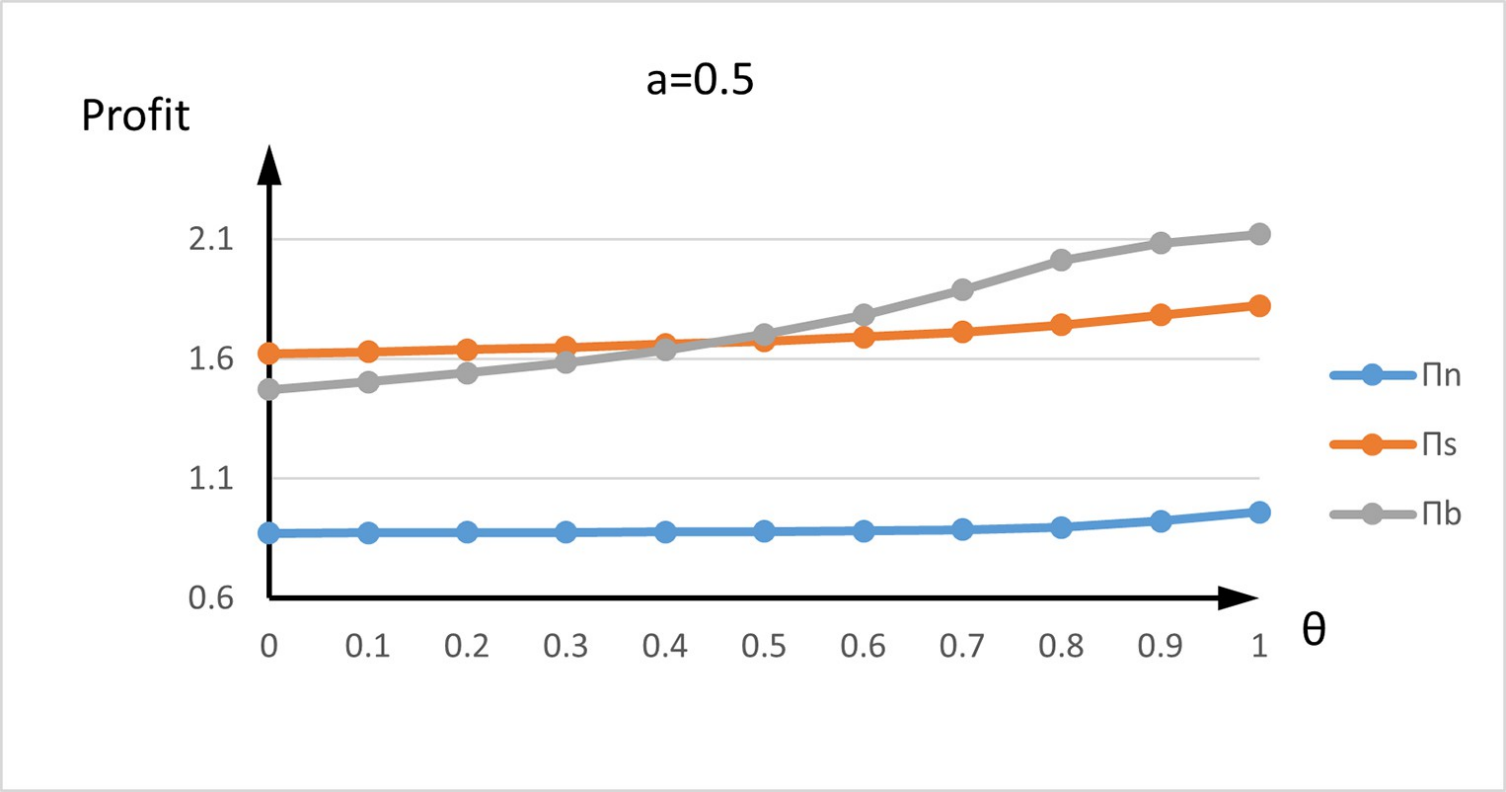
the influence of new high-type customers numbers on the profit of different trade-in strategies.

We now examine the impact of the high-value customer proportion and new customer number on profit in different trade-in strategies. Fig 3 depicts the seller’s expected total profits in three strategies, where *θ*-axis is the demand of all new high-type customers, and the profit-axis is the seller’s expected total profit under different trade-in strategies. The grey curve represents the seller’s expected total profit *π*^*n*^ with just advance selling no trade-in strategy, the orange curve represents the seller’s expected total profit *π*^*s*^ with advance selling and just trade-in strategy in second period, and the blue curve represents the seller’s expected total profit *π*^*b*^ with advance selling and trade-in strategy in both periods. Fig 3 shows that the profits of three trade-in strategies always strictly increase with *θ*. The two trade-in strategies are much better than no trade-in strategy for any *θ*, so either trade-in strategy is always optimal for the seller because the seller can set a higher pre-order price with a higher value demand. It is also noted that there exists a threshold value of *θ*′ which is the intersection of the two profit curves of strategy 2 and strategy 3, and the profit is the same as *θ* = *θ*′. The profit of strategy 3 is greater than strategy 2 as *θ*<*θ*′, and the profit of strategy 2 is greater than that of strategy 3 as *θ*>*θ*′. This means more new high-type customers do not always benefit the seller under strategy 3. Because more new high-type customers buy new generation products at the optimal pre-order price in the first period, the seller can earn more profit.

## 6. Discussion and conclusion

Although the trade-in strategy and advance selling strategy have been widely studied separately in extant literature. Our paper is one of few literature combined trade-in with advance selling strategy. And we adopt trade-in strategy as part of the advance selling strategy. In practice, many sellers including retailers and manufacturers have adopted the trade-in with advance selling strategies. However the trade-in rebate can hurt the seller’s profit and the discarded old products can damage the living environment. So designing an appropriate trade-in strategy is crucial for sellers that frequently release the new generation products.

In this study, based on the rational expectation equilibrium, we adopt dynamic programming to construct a two-period pricing model with three different trade-in strategies between the seller and consumers. We developed an analytical model to study how the innovation level, discount factor, and trade-in customers’ demand influence the choice of optimal trade-in strategy for advance selling. From the firm’s perspective, we identified that the seller can always make the highest pre-order price under the third strategy, i.e. offering trade-in in both periods because of old high-value strategic consumers. Given the innovation level, the seller can obtain the optimal discount factor, and both trade-in strategies are always more profitable than the no trade-in strategy for advance selling for ***θ*** and 03bb *λ*. Moreover, how the seller chooses the optimal trade-in strategy depends on the threshold value of new customer demand and trade-in demand. We helped the seller find the threshold value *θ*′ of new high-value customers under strategy 3. More new customers can hurt the seller when the new high-type customers are less than the threshold value. So we find that the optimal price is determined by stochastic factors such as the proportion of old customers, discount factor and product innovation level, which can help the seller make an appropriate decision of whether or not, and when to provide a trade-in rebate to old customers also depends on these parameters.

This study only focuses on the advance selling period and the regular selling period. The next avenue is to extend the period to a third phase when the product reaches the end-of-life cycle. We mainly take innovative products into account. In practice, many large e-commerce retailers such as Jindong, Suning, Taobao have adopted trade-in strategy with advance selling for durable products. Thus future related research can also pay attention to durable products such as washing machine, refrigerator, etc. The limitation of this study is that the trade-in rebate is constant and proportional to the pre-order or regular price. In future research, it can also be considered that the seller can adjust the trade-in rebate according to the characteristics of the old product. We just combined the trade-in strategy with advance selling between the seller and consumers. So how to choose supply chain contracts can be studied with trade-in and advance selling strategy between the supplier and retailer in the future. And how to stimulate product returns by designing return incentives and optimizing pricing strategies in this type of closed-loop supply chain would be interesting to explore.
